# Supplementary material for: Discovering Neurophysiological Characteristics of Pathological High-Frequency Oscillations in Epilepsy with an Explainable Deep Generative Model
Source: medRxiv. 2024 Jul 11:2024.07.10.24310189. Preprint. [Version 1] doi: 10.1101/2024.07.10.24310189 (PMC11261948; doi:10.1101/2024.07.10.24310189)
Supplement: Supplementary file 1 [file NIHPP2024.07.10.24310189v1-supplement-1.pdf]

medRxiv preprint doi: https://doi.org/10.1101/2024.07.10.24310189; this version posted July 11, 2024. The copyright holder for this preprint (which was not certified by peer review) is the author/funder, who has granted medRxiv a license to display the preprint in perpetuity.
It is made available under a CC-BY 4.0 International license .

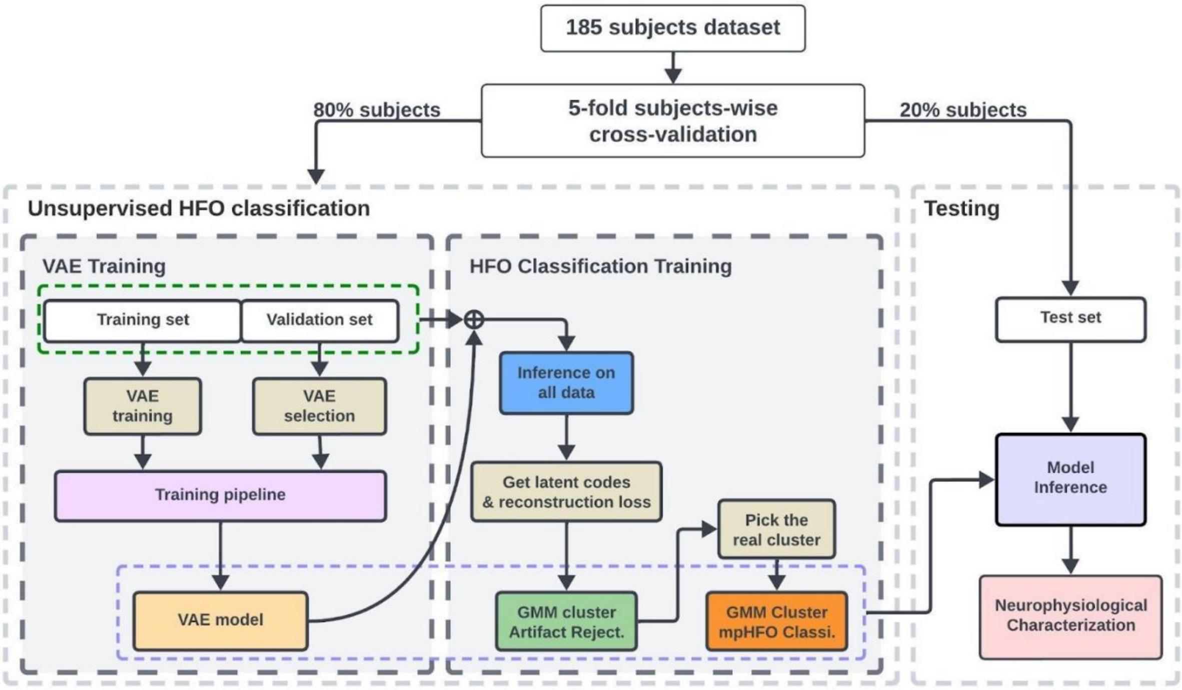

Supplementary Figure 1. VAE training and HFO classification workflow.

medRxiv preprint doi: https://doi.org/10.1101/2024.07.10.24310189; this version posted July 11, 2024. The copyright holder for this preprint (which was not certified by peer review) is the author/funder, who has granted medRxiv a license to display the preprint in perpetuity.
It is made available under a CC-BY 4.0 International license .

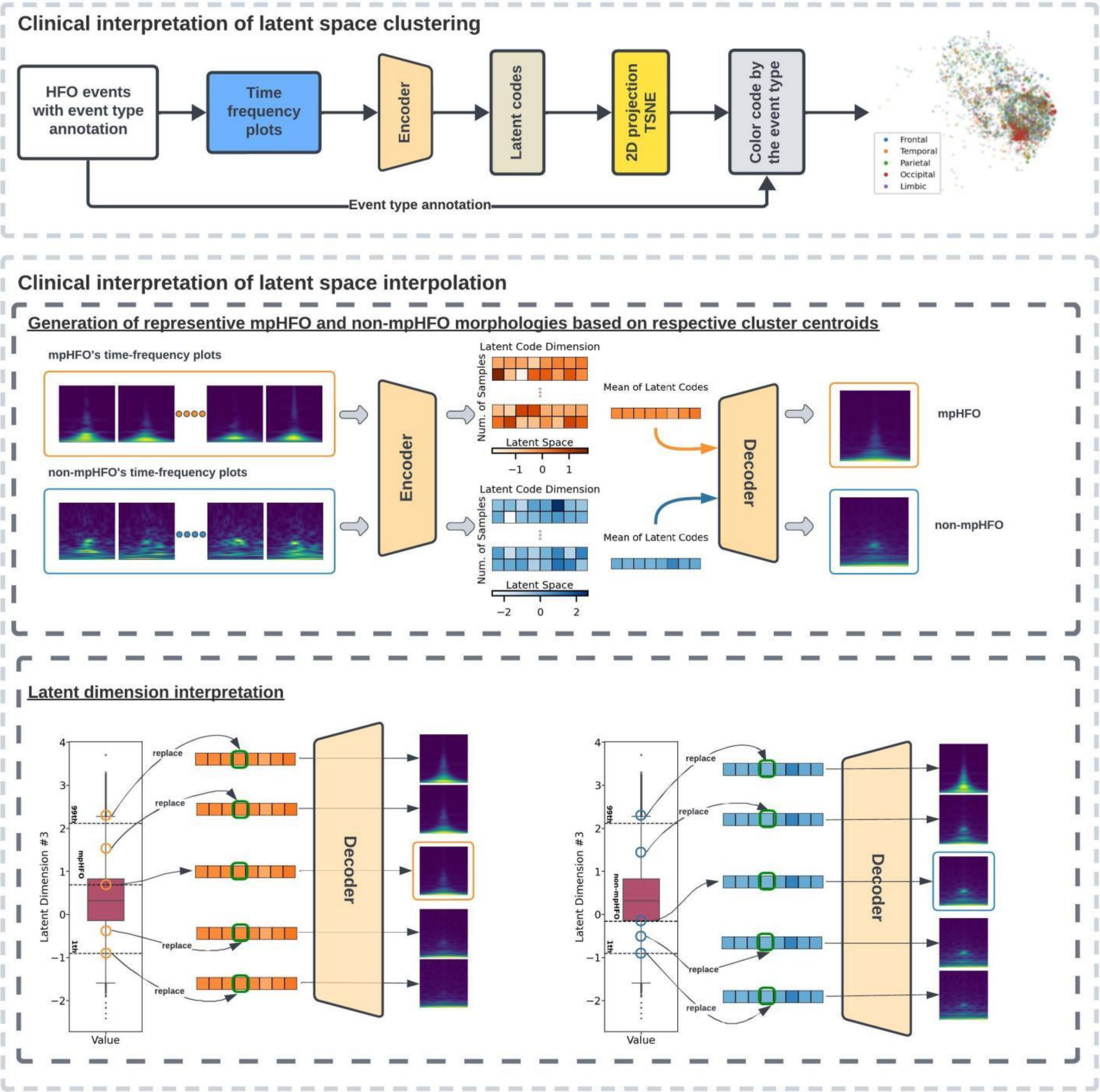

Supplementary Figure 2. Model interpretation analysis workflow.

medRxiv preprint doi: https://doi.org/10.1101/2024.07.10.24310189; this version posted July 11, 2024. The copyright holder for this preprint (which was not certified by peer review) is the author/funder, who has granted medRxiv a license to display the preprint in perpetuity.
It is made available under a CC-BY 4.0 International license .

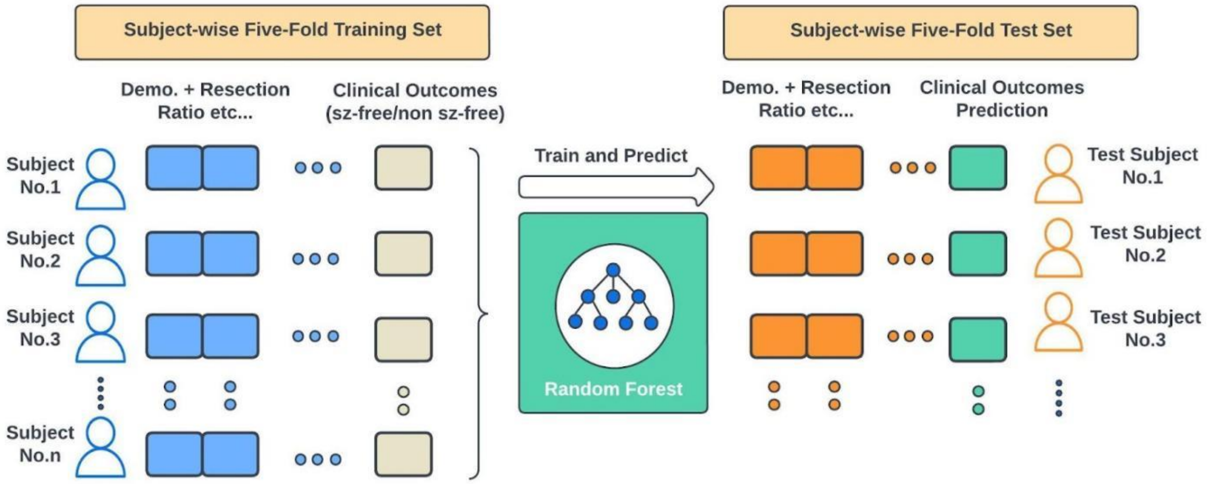

Supplementary Figure 3. Building postoperative seizure outcome prediction model using HFO information

medRxiv preprint doi: https://doi.org/10.1101/2024.07.10.24310189; this version posted July 11, 2024. The copyright holder for this preprint (which was not certified by peer review) is the author/funder, who has granted medRxiv a license to display the preprint in perpetuity.
It is made available under a CC-BY 4.0 International license .

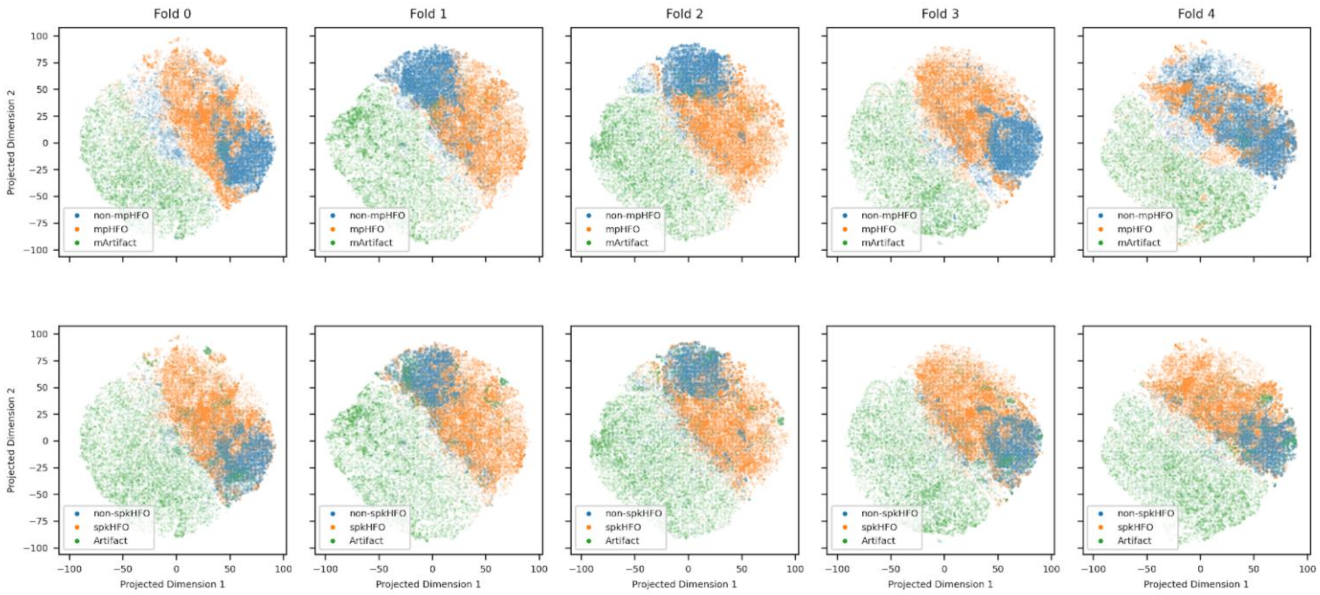

Supplementary Figure 4. Latent space comparison: This figure illustrates the 2D projection of the latent space, color-coded according to classifications from a VAE (mpHFO, non-mpHFO, and mArtifact) and from conventional supervised methods (spkHFO, non-spkHFO, and Artifact), evaluated across five folds. It highlights the alignment between VAE predictions and conventional supervised classifications, demonstrating that classifiers developed from unsupervised learning can achieve performance comparable to those developed from supervised methods.

medRxiv preprint doi: https://doi.org/10.1101/2024.07.10.24310189; this version posted July 11, 2024. The copyright holder for this preprint (which was not certified by peer review) is the author/funder, who has granted medRxiv a license to display the preprint in perpetuity.
It is made available under a CC-BY 4.0 International license .

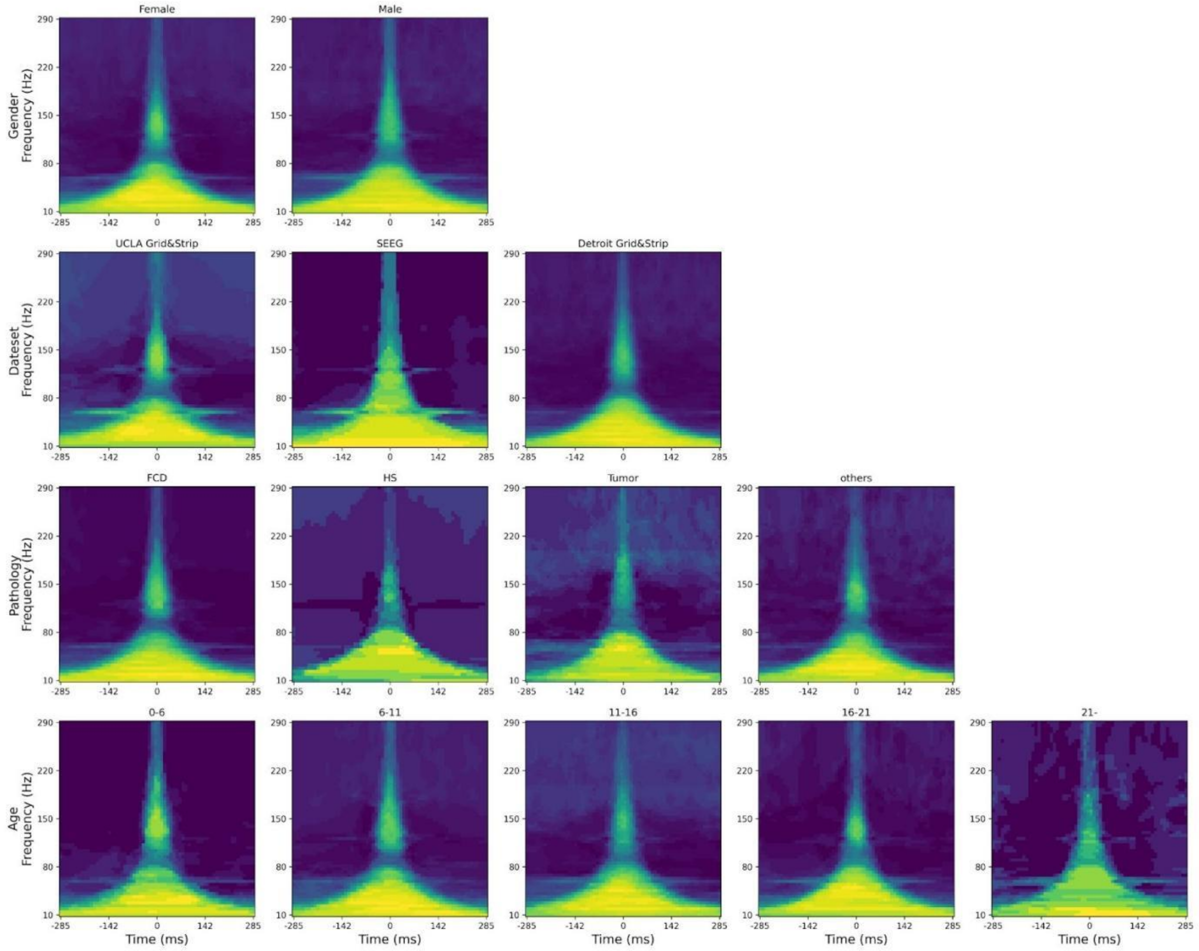

Supplementary Figure 5. mpHFO shares similar morphology across the variables. The morphological analysis of mpHFOs' time-frequency plot is shown based on subgroups, each corresponding to a specific variable. Consistent morphological features of mpHFOs are seen across the subgroups. mpHFOs have higher values throughout the HFO band (> 80 Hz), around the center point (0 ms, where HFOs were detected) than non-mpHFOs; furthermore, higher values of mpHFOs at the sub-HFO band (10-80 Hz) throughout the time window compared to non-mpHFOs are seen.

medRxiv preprint doi: https://doi.org/10.1101/2024.07.10.24310189; this version posted July 11, 2024. The copyright holder for this preprint (which was not certified by peer review) is the author/funder, who has granted medRxiv a license to display the preprint in perpetuity.
It is made available under a CC-BY 4.0 International license .

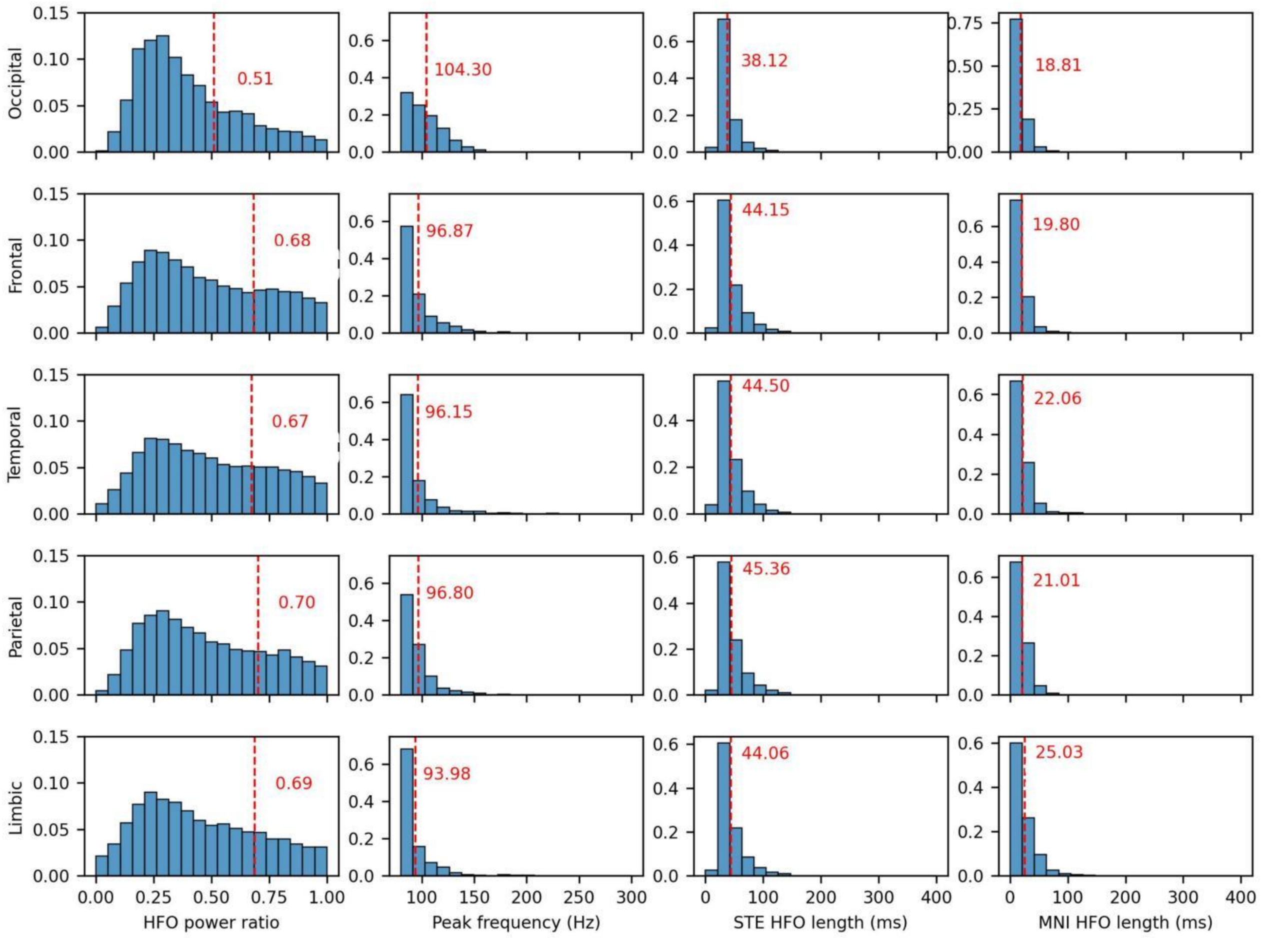

Supplementary Figure 6. Conventional physiological non-mpHFO characteristics across different anatomical locations. This figure displays multiple aspects of HFOs: the HFO power ratio in the first column, HFO peak frequency in the second column, STE HFO length in the third column, and MNI HFO length in the fourth column. The HFO power ratio is defined as the ratio of the signal power within ±50ms of the HFO center to the power within the entire time window of ±285ms. The peak frequency is identified as the frequency component that has the most power within the 80-300 Hz range. Both STE and MNI HFO lengths are measured in milliseconds as the duration of the HFO event. Each histogram's mean value is annotated within its respective panel. Additionally, statistical comparisons revealed that physiological HFOs in the occipital region exhibited lower power ($p<0.001$, one-sided t-test), higher peak frequency ($p<0.001$, one-sided t-test), and shorter durations in both STE and MNI HFOs ($p<0.001$ for all, except in the frontal location where $p<0.01$, one-sided t-test) compared to those in other anatomical locations.

.

medRxiv preprint doi: https://doi.org/10.1101/2024.07.10.24310189; this version posted July 11, 2024. The copyright holder for this preprint (which was not certified by peer review) is the author/funder, who has granted medRxiv a license to display the preprint in perpetuity.
It is made available under a CC-BY 4.0 International license .

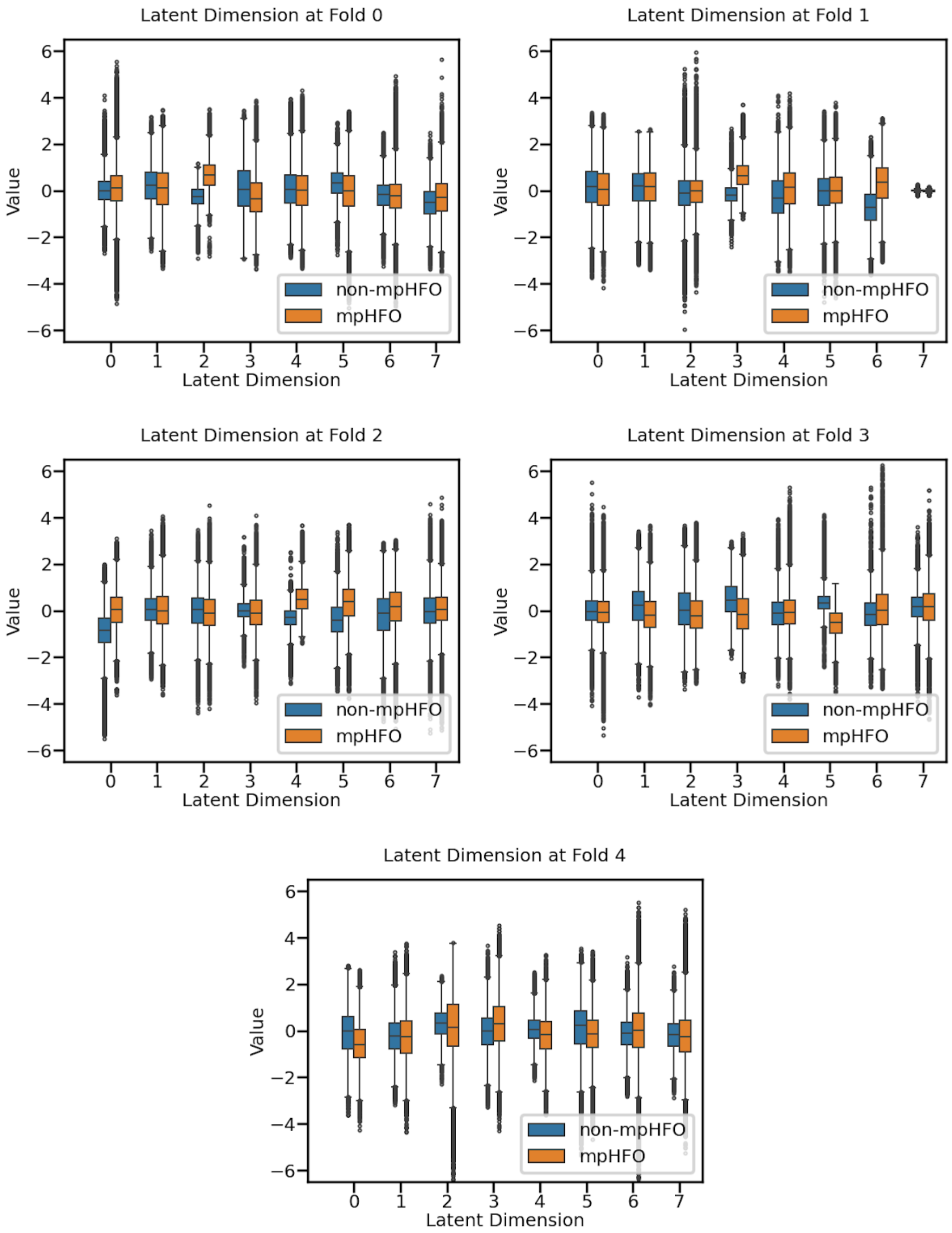

Supplementary Figure 7. Full latent space visualization across five folds. This visualization presents the distribution of each latent dimension, trained within five folds and depicted by separating mpHFO (in orange) and non-mpHFO (in blue). It is evident that mpHFO and non-mpHFO exhibit significant separation in some dimensions while others show less distinction.

medRxiv preprint doi: https://doi.org/10.1101/2024.07.10.24310189; this version posted July 11, 2024. The copyright holder for this preprint (which was not certified by peer review) is the author/funder, who has granted medRxiv a license to display the preprint in perpetuity.
It is made available under a CC-BY 4.0 International license .

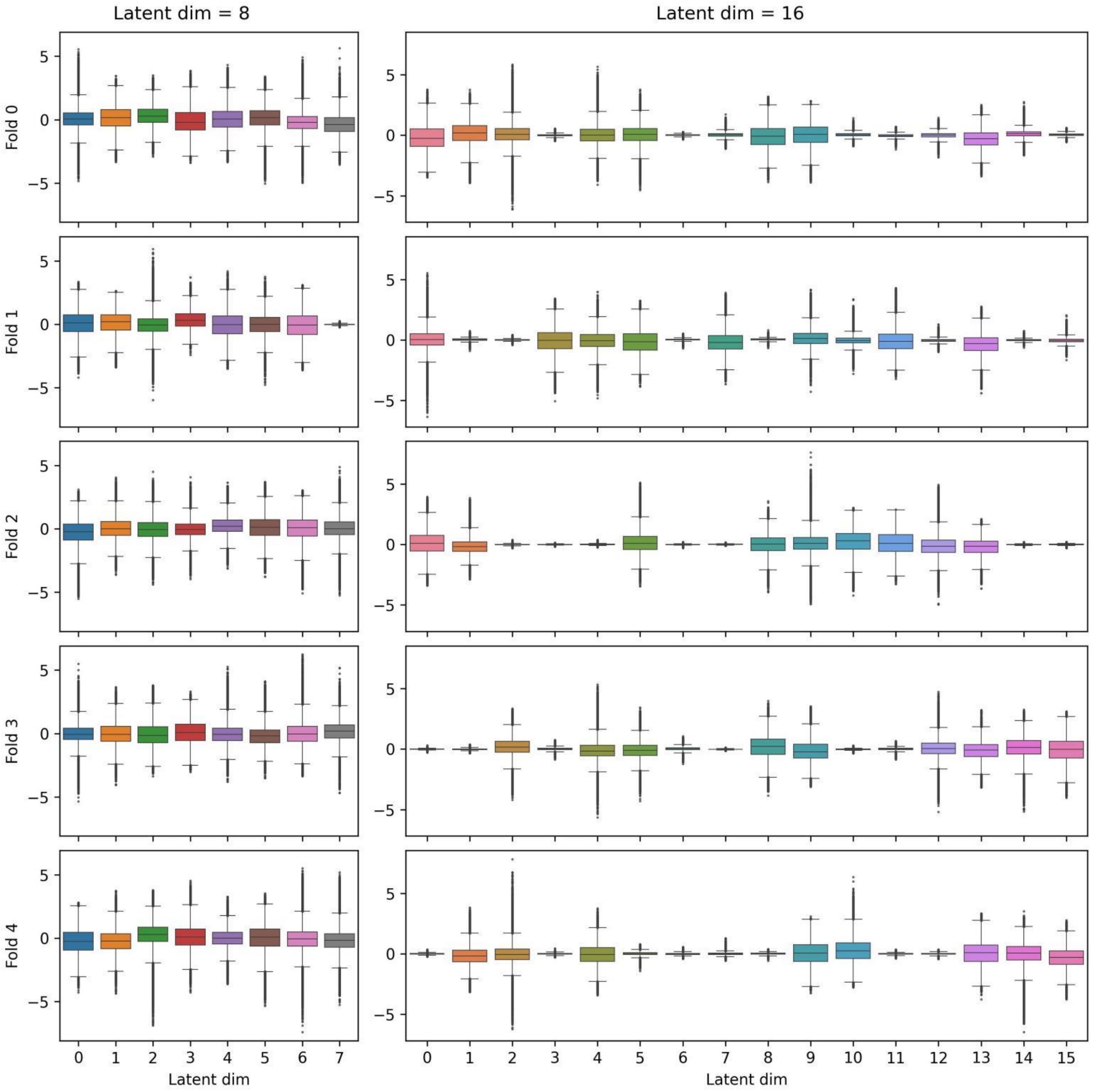

Supplementary Figure 8. Ablation study on different latent dimensions. The left panel shows the distribution of dimensions for a VAE model trained with a latent dimension of 8, across five folds. The right panel displays the distribution for the same model configuration but with a latent dimension of 16. Several dimensions in the 16-dimension model do not spread well, indicating that these dimensions are underutilized during training. This compactness suggests a potential reduction in dimensionality. Based on these observations and the more effective utilization of dimensions seen in the boxplot for the 8-dimension model, we chose to proceed with a latent dimension of 8, as it demonstrates optimal use of all available dimensions during training.

medRxiv preprint doi: https://doi.org/10.1101/2024.07.10.24310189; this version posted July 11, 2024. The copyright holder for this preprint (which was not certified by peer review) is the author/funder, who has granted medRxiv a license to display the preprint in perpetuity.
It is made available under a CC-BY 4.0 International license .

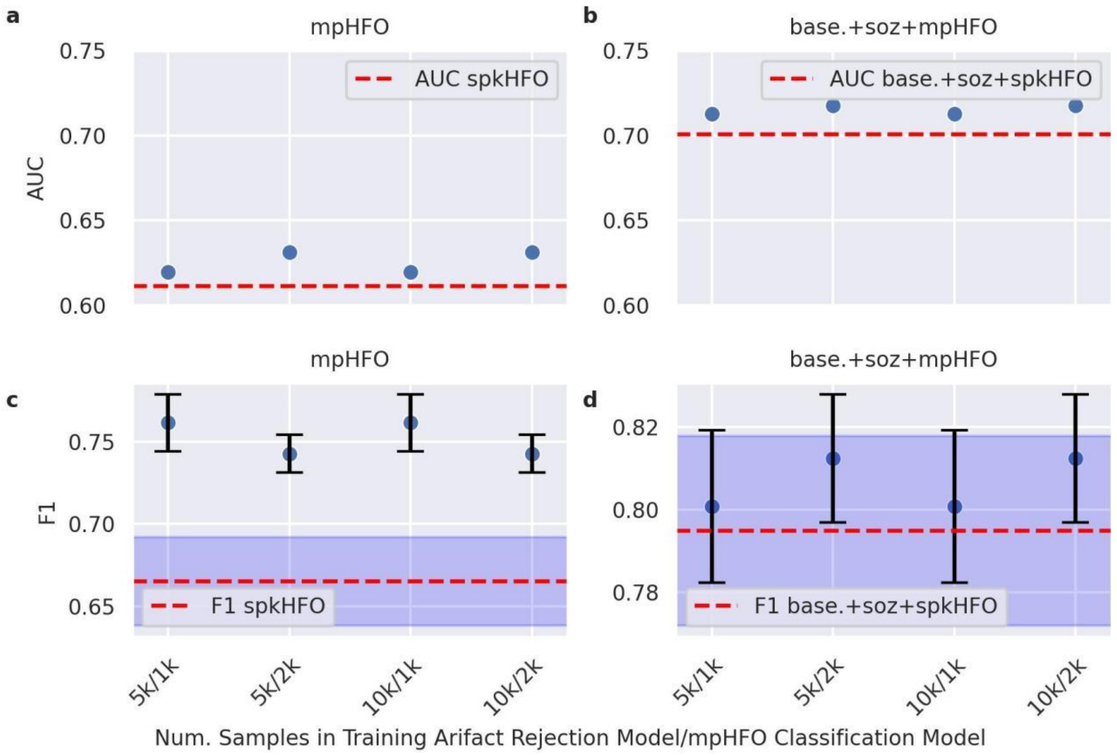

This is the new figure using threshold = 0

Supplementary Figure 9. Ablation study on predicting surgical outcomes using different sample sizes for training GMM models. We conducted experiments using different stratified sampling to train GMM models and investigated how capping the number of samples per subject used to train the artifact rejection and mpHFO classification impacts surgical outcomes prediction performance metrics such as AUC and F1 score. We compared models capped at 10,000 or 5,000 samples per subject for artifact rejection and 2,000 or 1,000 samples for mpHFO classification. (a) Comparison of AUC scores for mpHFO models trained from different stratified sampling sizes against those from spkHFO models. (b) A similar comparison for base.+soz+mpHFO against base.+soz+spkHFO. (c) Comparison of F1 scores for mpHFO models trained from different stratified sampling sizes against those from spkHFO models. (d) Similar procedure as in (c), Similar comparison for base.+soz+mpHFO against base.+soz+spkHFO.

medRxiv preprint doi: https://doi.org/10.1101/2024.07.10.24310189; this version posted July 11, 2024. The copyright holder for this preprint (which was not certified by peer review) is the author/funder, who has granted medRxiv a license to display the preprint in perpetuity.
It is made available under a CC-BY 4.0 International license .

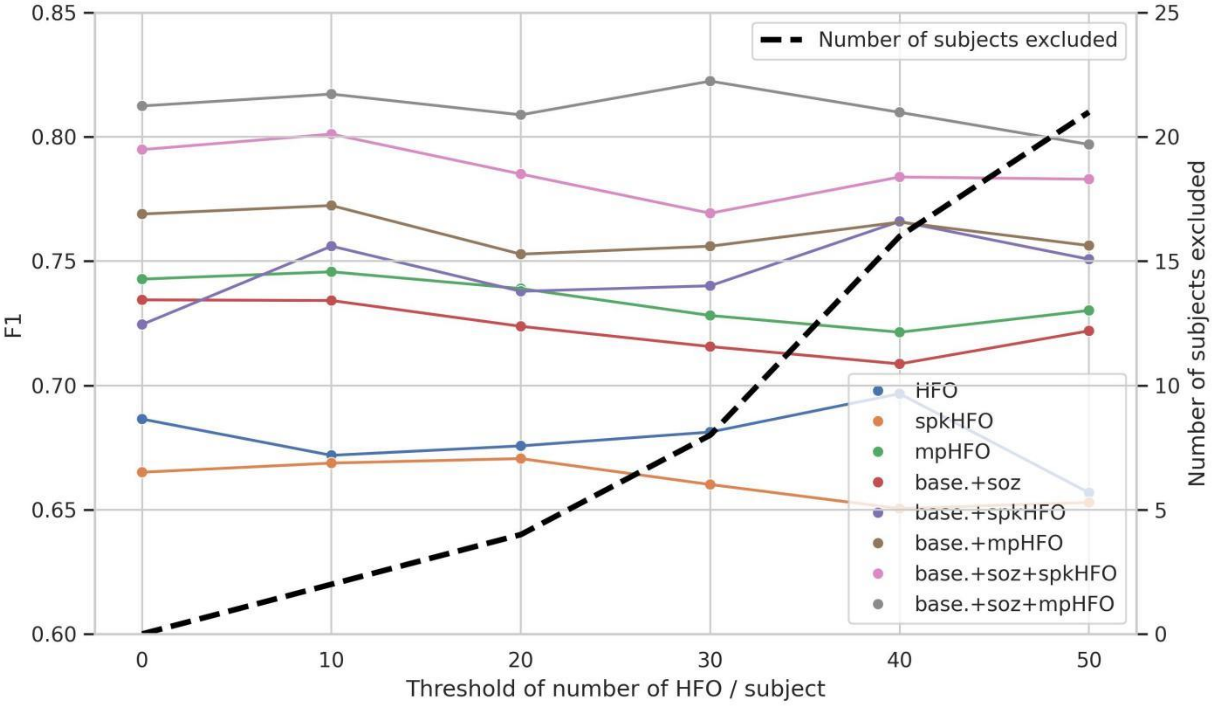

Supplementary Figure 10. Ablation study on predicting surgical outcomes by excluding patients with fewer HFOs: We conducted an ablation study to assess the impact of excluding patients with a smaller number of high-frequency oscillations (HFOs) on the performance of our Random Forest model in predicting post-surgical outcomes. In addition to the results presented in the main manuscript, which included all patients, we compared the F1 scores for predicting post-surgical outcomes by selectively including patients with a minimum of 10, 20, 30, 40, and 50 HFOs. The F1 scores were plotted for different feature sets used in constructing the Random Forest model. The results indicate that the F1 score remains consistent even when including only patients with more than 50 HFOs, demonstrating the robustness of our self-supervised framework.

medRxiv preprint doi: https://doi.org/10.1101/2024.07.10.24310189; this version posted July 11, 2024. The copyright holder for this preprint (which was not certified by peer review) is the author/funder, who has granted medRxiv a license to display the preprint in perpetuity.
It is made available under a CC-BY 4.0 International license .

Supplementary Table 1

Dataset	No. Event	No. Artifact	No. spkHFO	No. non-spkHFO
STE Detector				
Detroit grid/strip	35610	4374	20481	10755
UCLA grid/strip	103360	14578	54455	34327
UCLA SEEG	35861	7886	21748	6227
All STE HFOs	174831	26838	96684	51309
MNI Detector				
Detroit grid/strip	36279	6494	26953	2832
UCLA grid/strip	124064	42848	69266	11950
UCLA SEEG	351236	275550	50434	25252
All MNI HFOs	511579	324892	146653	40034

medRxiv preprint doi: https://doi.org/10.1101/2024.07.10.24310189; this version posted July 11, 2024. The copyright holder for this preprint (which was not certified by peer review) is the author/funder, who has granted medRxiv a license to display the preprint in perpetuity.
It is made available under a CC-BY 4.0 International license .

Supplementary Table 2

Feature (s)	Accuracy	F1
HFO	0.559(0.089)	0.686(0.081)
spkHFO	0.553(0.141)	0.665(0.134)
mpHFO	0.657(0.051)	0.743(0.056)
base.+ soz	0.637(0.106)	0.734(0.090)
base.+ spkHFO	0.624(0.113)	0.725(0.108)
base.+ mpHFO	0.668(0.111)	0.769(0.096)
base.+ soz + spkHFO	0.719(0.126)	0.795(0.115)
base.+ soz + mpHFO	0.732(0.084)	0.812(0.078)

medRxiv preprint doi: https://doi.org/10.1101/2024.07.10.24310189; this version posted July 11, 2024. The copyright holder for this preprint (which was not certified by peer review) is the author/funder, who has granted medRxiv a license to display the preprint in perpetuity.
It is made available under a CC-BY 4.0 International license .

Supplementary Figure 3

	STE
Filter freq (Hz)	[80, 300]
RMS window (s)	0.003
Min window (s)	0.006
Min gap (s)	0.01
Min osc (count)	6
RMS thres (in SD)	5
Peak thres (in SD)	3
Epoch len (ms)	600

	MNI
Filter freq (Hz)	[80, 300]
Epo CHF (s)	60
Per CHF (%)	0.95
Min win (s)	0.01
Min gap (s)	0.01
Thrd perc (%)	0.999999
Base seg (s)	0.125
Base shift (0-1)	0.5
Base thrd (0-1)	0.67
Base min (%)	5
Epoch time (ms)	10
Seed	0
